# Stylet Morphometrics and Citrus Leaf Vein Structure in Relation to Feeding Behavior of the Asian Citrus Psyllid *Diaphorina citri*, Vector of Citrus Huanglongbing Bacterium

**DOI:** 10.1371/journal.pone.0059914

**Published:** 2013-03-26

**Authors:** El-Desouky Ammar, David G. Hall, Robert G. Shatters

**Affiliations:** United States Department of Agriculture-Agricultural Research Service, Horticultural Research Laboratory, Fort Pierce, Florida, United States of America; United States Department of Agriculture, United States of America

## Abstract

The Asian citrus psyllid (ACP), Diaphorina citri (Hemiptera: Psyllidae), is the primary vector of the phloem-limited bacterium *Candidatus* Liberibacter asiaticus (LAS) associated with huanglongbing (HLB, citrus greening), considered the world’s most serious disease of citrus. Stylet morphometrics of ACP nymphs and adults were studied in relation to citrus vein structure and to their putative (histologically verified) feeding sites on Valencia orange leaves. ACP nymphs preferred to settle and feed on the lower (abaxial) side of young leaves either on secondary veins or on the sides of the midrib, whereas adults preferred to settle and feed on the upper (adaxial) or lower secondary veins of young or old leaves. Early instar nymphs can reach and probe the phloem probably because the distance to the phloem is considerably shorter in younger than in mature leaves, and is shorter from the sides of the midrib compared to that from the center. Additionally, the thick-walled ‘fibrous ring’ (sclerenchyma) around the phloem, which may act as a barrier to ACP stylet penetration into the phloem, is more prominent in older than in younger leaves and in the center than on the sides of the midrib. The majority (80–90%) of the salivary sheath termini produced by ACP nymphs and adults that reached a vascular bundle were associated with the phloem, whereas only 10–20% were associated with xylem vessels. Ultrastructural studies on ACP stylets and LAS-infected leaves suggested that the width of the maxillary food canal in first instar nymphs is wide enough for LAS bacteria to traverse during food ingestion (and LAS acquisition). However, the width of the maxillary salivary canal in these nymphs may not be wide enough to accommodate LAS bacteria during salivation (and LAS inoculation) into host plants. This may explain the inability of early instar nymphs to transmit LAS/HLB in earlier reports.

## Introduction

The Asian citrus psyllid (ACP), Diaphorina citri Kuwayama (Hemiptera: Psyllidae), is the primary vector of the phloem-limited bacteria (*Candidatus* Liberibacter spp.) associated with huanglongbing (HLB, or citrus greening), now considered the world’s most serious disease of citrus [1], [2]. ACP and HLB were first discovered in Asia, but one or both are presently distributed in the Southern region of the United States, the Caribbean, Mexico, Central and South America, Africa, and several countries in South Asia, the Middle East, Réunion and Mauritius islands [3]. Ca. L. asiaticus (LAS) is the putative causal agent of HLB in most of the New World and Asia [2].

LAS is transmitted in a persistent manner by both nymphs and adults of ACP, and infected individuals may retain infectivity throughout their lives [4], [5], [6]. LAS has been detected either by quantitative polymerase chain reaction (qPCR) analyses or fluorescent *in situ* hybridization in several tissues of ACP including the midgut, salivary glands, hemolymph and fat tissues [7], [8]. Inoue et al. [9] reported that LAS multiplies in ACP nymphs but not in the adults, and suggested that this may account for the higher rate of LAS transmission by nymphs compared to that of the adults. Interestingly, third to fifth instar nymphs of ACP are efficient in acquiring and inoculating LAS from diseased to healthy citrus plants, but younger (1^st^ and 2^nd^) instars reportedly cannot acquire and/or inoculate LAS [4], [5], [6]. Normally, low inoculation rates by *D. citri* adults (1.3–12.2%) have been reported [10], [11], [12], and Ammar at al. [7], [8] suggested that the midgut and salivary glands may act as barriers to LAS transmission by ACP.

In Hemiptera, feeding structures (stylets and other mouthparts) and feeding behavior are very important in the processes of acquisition of various disease agents from diseased plants and their subsequent inoculation into healthy plants [13], [14], [15]. The piercing-sucking mouth parts of ACP adults and their salivary sheaths have been studied [16], [17], but those of nymphs have not been studied so far. Also, the feeding behavior of ACP adults, but not nymphs, has been studied with electrical penetration graphs (EPG) [16], [18]. ACP adults were found to ingest sap mainly from the phloem sieve elements, although occasionally they also appear to ingest sap from xylem vessels [16], [18]. Cen et al. [18] compared EPG waveforms of ACP adults feeding on healthy and LAS-infected ACP. He reported that in HLB-diseased plants, the times to first and sustained salivation in the phloem were longer than those in healthy plants, and that as HLB symptom expression increased, the percentage of time spent by ACP salivating during the phloem phase increased but the percentage of time spent in all phloem activities (i.e. salivation and ingestion) were reduced gradually. In contrast, the percentage of time spent on xylem activities increased as did the proportion of psyllids ingesting from xylem in diseased plants. Probably because ACP nymphs are smaller, more fragile and thus more difficult to handle, no EPG or other studies on the feeding behavior of nymphs have been reported.

ACP females oviposit their eggs on young terminal citrus tissue including leaf folds, petioles, auxiliary buds, upper and lower surfaces of young leaves, and tender stems [19]. Early instar nymphs are docile and move only when disturbed or overcrowded [19], whereas older nymphs and adults are more mobile. First to third instar nymphs cannot develop on mature leaves, whereas survival and adult emergence of 3^rd^ to 5^th^ instar nymphs were significantly higher when reared on younger citrus leaves compared to those reared on older ones [3], [19], [20]. ACP nymphs and adults are known to settle and feed for long periods on citrus leaves assuming certain feeding postures, with the adults having about 40° angle with the leaf surface and the nymphs staying almost motionless and flat for a long period [3], [16], [18], [19].

The present work was done with the following objectives: A. study the stylet length of ACP nymphs and adults in relation to their feeding behavior and to the structure of citrus leaf veins; B. compare the putative feeding sites of ACP nymphs and adults (settling sites later verified to be feeding sites via microscopy of extended stylets and/or histology of the leaves) on young/old and healthy/LAS-infected citrus leaves; and C. study the width of the maxillary food and salivary canals in ACP adults and 1^st^ instar nymphs in relation to the diameter of LAS bacterium, to see whether the food and/or salivary canals in the maxillary stylets can be a barrier to LAS acquisition and/or inoculation by early instar ACP nymphs.

## Materials and Methods

### Insects and Host Plants

Healthy (non-HLB infected) nymphs and adults of ACP (*D. citri*) were taken from our laboratory colony that has been maintained for several generations on young healthy citrus plants (*Citrus macrophylla* Wester) in the greenhouse. Individuals from the colony were assayed with quantitative polymerase chain reaction (qPCR) tests [12] every three months to ensure that the colony remained free of *Ca.* Liberibacter asiaticus (LAS) associated with HLB. Healthy (non-HLB infected) citrus leaves were taken from uninfected sweet orange trees [*Citrus sinensis* (L.) Osbeck] var. ‘Ridge Pineapple’ or ‘Valencia’, or from *C. macrophylla* trees, all grown from healthy seeds in the greenhouse. HLB-infected leaves were taken from greenhouse ‘Valencia’ trees infected with LAS several months earlier and tested positive for LAS in qPCR tests.

### Maintenance and Behavioral Observations of ACP Nymphs and Adults

**Figure 1:**
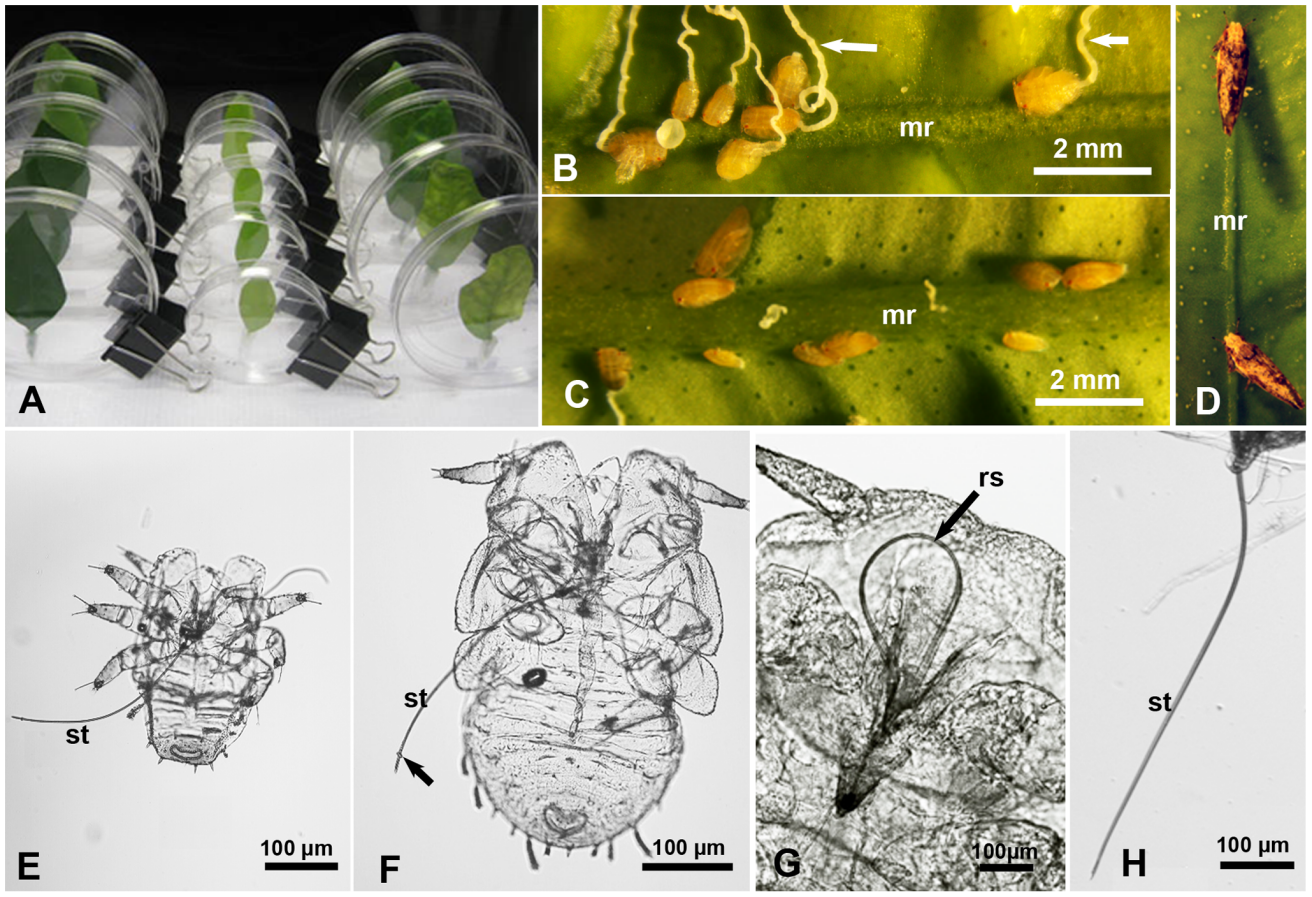
Studying feeding sites and stylets of *Diaphorina citri* nymphs and adults. **A.** Vertically held Petri dishes used for studying feeding sites of psyllid adults on Valencia orange leaves**. B & C.** Psyllid nymphs in their settling/feeding posture, mainly on the sides, rather than top, of the midrib (mr) on the upper (B) or lower (C) leaf surface; arrows indicate waxy honeydew excretions. D. Psyllid adults in their typical feeding posture, on the upper midrib (mr) of a citrus leaf. **E & F**. Exuviae of 1^st^ and 2^nd^ instar nymphs, respectively, with fully extended stylets (st); arrow indicates salivary flange around the stylet bundle. **G**. Psyllid nymph with retracted/looped stylets (rs). **H**. Extended stylets (st) of an adult psyllid.

Putative feeding sites of ACP nymphs and adults on citrus leaves were observed using a stereomicroscope (Leica MZ16) fitted with a Leica DFC 320 camera, or using another stereomicroscope (Leica M60) fitted with a video camera (Leica DFC290 HD) (Leica, Switzerland). ACP adults of both sexes were caged in groups (5–10/group) on excised young citrus leaves. The cut end of the petiole was placed in a small (0.5 ml) micro-centrifuge tube filled with water. Each leaf was then placed in a 50-ml polypropylene tube (Fisher Scientific, Pittsburg, PA) as described earlier [20], [21] or in a Petri dish for easier observation under the stereomicroscope (Fig. 1A). Additionally, young terminal shoots of *C. macrophylla* with young ACP nymphs were similarly placed in water-filled microfuge tubes and caged in 50-mL tubes or Petri-dishes as described above. With both nymphs and adults, the caging tubes or Petri-dishes were placed on the bench top in the laboratory (at 23.7±1.5°C) with 14 hr light per day (1 Fluorescent, 4200 k bulb, ca. 50 cm above). Identification of various nymphal instars of *D. citri* followed the drawings by Catling [22].

ACP nymphs or adults that settled and assumed their respective feeding postures mentioned above (Figs. 1 B-D) on various sites of the leaf (midrib or smaller veins on either side of the leaf), were considered to be putatively feeding. Previous observations showed that when ACP nymphs or adults were immobilized while assuming these postures (using chloroform vapor or freezing for a few minutes) their stylets were always fully or partially extended, which indicates that they were feeding. Additionally, histology of citrus leaves showed that salivary sheaths were found at or close to the presumed (putative) feeding sites, as elucidated below in the Methods and Results sections.

### Measuring the Stylets and Body Length of Nymphs and Adults

As we reported earlier [21], the exuviae of various nymphal instars in ACP and many other hemipterans have fully extended stylets that can be studied morphologically more readily than those of live nymphs (Figs. 1E–G). Thus, exuviae from the five nymphal instars of ACP (normally still attached to the plant parts on which they were feeding during molting) were pulled out gently from these plant parts using fine forceps (Fontax no. 5; Electron Microscopy Sciences, Washington, PA, USA). To study the stylet- and body-length in various nymphal instars, ACP exuviae were mounted on glass slides with 50–80% glycerol in water, examined by confocal or light microscopy at 40–100X. However, to study the stylet length in ACP adults, live males and females were caged for 1–2 days on young citrus leaves in Petri-dishes (5–10 insects/dish). These insects were immobilized during feeding by placing a piece of filter paper soaked in chloroform inside each dish for 5–10 min in a fume hood. Under a stereomicroscope, the insect was then removed (with its stylets still fully extended) by gently pulling it out with fine forceps from the leaf. Subsequently, the head (including extended stylets) was separated from the thorax with a razor, fixed (2 h to overnight) in 4% paraformaldehyde in phosphate buffered saline (PBS, pH 7.4, Polysciences, Warrington, PA), washed in PBS twice, then mounted in 50–80% glycerol on microscope slides, and finally examined by confocal or light microscopy (Fig. 1H). With both nymphs and adults, the images obtained for the extended stylets were analyzed with the ‘ImageJ’ computer program (http://rsb.info.nih.gov/ij/).

### Feeding Sites of ACP on Healthy and LAS-infected Citrus Leaves

Healthy young ACP adults (within 1 wk after adult emergence) were caged on the following leaves of ‘Valencia’ orange in plastic Petri-dishes for 8–9 days (10 adults/leaf/dish; 5 dishes/treatment): A. young healthy leaves, B. old healthy leaves, C. young LAS-infected leaves, and D. old LAS-infected leaves. All leaves were fully unfolded, but the ‘young’ leaves (healthy and infected) were 25–35 mm wide and 54–59 mm long, whereas ‘old’ leaves (of both categories) were 44–45 mm wide, and 62–91 mm long. Most of the infected leaves used showed symptoms of HLB [2], [3]. The petioles of all leaves were placed in water-filled microfuge tube as described above. The young leaves were then placed in small Petri-dishes (90 mm wide) whereas old leaves were placed in larger dishes (140 mm wide). All dishes were placed vertically (Fig. 1A) on the bench top in the laboratory (at 23.7±1.5°C) with 14 hr light per day. Each leaf was examined twice daily for 9 days, at 8.30 am and 4.30 pm, to record the number of adults in a typical feeding posture (Fig. 1D) on the midrib or smaller veins, on both the upper (adaxial) and lower (abaxial) sides of the leaf. This experiment was repeated twice, and in the second time, the leaves used and the adults that fed on these leaves were tested by qPCR, using HLBaspr primers, as described by Ammar et al. [12], to see if these adults acquired LAS from infected leaves during the caging period of the experiment. Similar experiments were done using ACP nymphs that hatched from eggs laid on the terminal shoots of young healthy or LAS-infected ‘Valencia’ leaves. To minimize any possible damage to the stylets if nymphs were transferred to new leaves/plants, the putative feeding sites of 3^rd^–5^th^ instar nymphs were studied on the youngest 7–8 unfolded leaves of the same shoots on which these nymphs had hatched earlier. Because nymphs are much more sessile than adults, the putative feeding sites of nymphs were observed daily (rather than twice a day) for 4 consecutive days. The number of nymphs observed daily ranged from 80 to 251/treatment/experiment.

### Preparing Citrus Leaf Sections to Study Vein Structure and ACP Salivary Sheaths

ACP nymphs or adults were caged for 7 days on terminal shoots or young Valencia orange leaves, respectively, in Petri-dishes (≥10 insects/dish), then immobilized while putatively feeding as described above. Under a stereomicroscope, a clean razor blade was used to cut a small piece (ca. 2–3 mm long) of the plant tissue around the putative feeding position of each insect, normally including the midrib or other veins with the surrounding tissue, before removing the immobilized insect. The plant piece was immediately transferred to a drop of PBS or the fixative (4% paraformaldehyde in PBS) on a clean glass slide, and sectioned by hand using a sharp razor blade to the thinnest possible sections under a stereomicroscope (at 20x or higher). These sections (determined by confocal microscopy to be ca. 50–70 µm thick) were quickly transferred to microfuge tubes with the fixative for 1–7 days, before washing 3 times with PBS-T (PBS with 0.1% Triton×100). They were then stained for 5 min with the nuclear fluorescent stain propidium iodide, if they were to be examined by confocal microscopy, or not stained if to be examined only by epifluorescence microscopy. If stained, sections were washed again in PBS-T three times, before mounting on microscope slides using Fluoro-Gel (antifade mounting medium, Electron Microscopy Sciences, Hatfield, PA, USA). Mounted sections were kept in the dark at 4°C until examination with UV light using an epifluorescence inverted microscope (Olympus IX70, with 4X, 10X or 20X objectives) fitted with a camera system. An average of 20 sections (10–29) were examined from each of the eight leaf categories tested (combinations of healthy, diseased, young, and old leaves). These sections were examined mainly using autofluorescence (with no filter cubes), sometimes also with transmitted light in order to show the cell boundaries in the plant tissues examined. Some plant sections (stained with propidium iodide) were also examined with a confocal laser scanning microscope (Zeiss LSM 510, 10x or 20x objectives, He/Ne laser) with excitation wavelength of 543, sometimes simultaneously with DIC (differential interference contrast). The distance to the phloem, phloem width, and other measurements were taken from photographed images of these sections using ‘ImageJ’ computer program. In all figures of plant sections shown here, the sections are shown with the upper (adaxial) side of the leaf up (or on upper right) to help in the orientation and interpretation of these figures.

### Transmission Electron Microscopy (TEM) of ACP Stylets and Citrus Leaves

ACP adults and 1^st^ instar nymphs were caged for 1–2 days on young citrus leaves in Petri-dishes (5–10 insects/dish) before being immobilized while feeding, as described above. The thorax-head part (including the extended stylets) of adults, and whole 1^st^ instar nymphs were then fixed in 3% glutaraldehyde in phosphate buffer, pH 7.4. Specimens were kept in this fixative for 4 h to overnight at 4°C, postfixed in 1% osmium tetroxide in the same buffer for 1–2 h at 4°C, washed in buffer, dehydrated in ethanol and propylene oxide, and embedded in Spurr’s resin, as previously described [23]. Small pieces (1–2 mm wide) of healthy or LAS-infected citrus leaves that included the midrib were similarly fixed and prepared for TEM. Semithin sections (1–2 µm thick**)** in leaves or stylets, cut with an ultra microtome (Leica EM UC7 RT), were stained with toluidine blue and examined by light microscopy at 40–200X. Ultrathin sections (cut with the same microtome) were stained with uranyl acetate and lead citrate and examined at 25 Kv using a scanning-transmission electron microscope (Hitachi S-4800, Hitachi, Pleasanton, CA) in the TEM mode, at magnifications of 400X to 30,000X. The width of the maxillary food and salivary canals was measured using ‘ImajeJ’ computer program, with images obtained from ultrathin sections of adult stylets (25 sections from 3 adults) and 1^st^ instar nymphs (34 sections from 4 nymphs).

### Statistical Analysis

Data means ± SEM were computed using PROC MEANS; analyses of variance were conducted using PROC ANOVA, PROC GLM or PROC TTEST (all procedures by SAS Institute, 2010). Percentage data were arcsine-transformed for statistical analyses [24]. All evaluations of statistical significance were conducted at *P*<0.05–0.0001. χ^2^ tests were performed on the ratios also with *P*<0.05–0.0001 (with df = 1).

## Results

### Stylet and Body Length in ACP Nymphs and Adults

The mean stylet length in the five nymphal instars of ACP ranged between 266.0 µm (83% of body length) in the 1^st^ instar to 614.5 µm (34% of body length) in the 5^th^ instar (Table 1, Figs. 1E–F). ANOVA indicated that differences between all five nymphal instars in the stylet length, body length and the ratio of stylet/body length were significant (*P* = 0.0001). However, the stylet length of 5^th^ instar nymphs was not significantly different from that of the adults (610.9 µm), although the ratio of stylet to body length was significantly smaller in the adults (24%) compared to that in 5^th^ instar nymphs (Table 1).

**Table 1:** Mean stylet length, body length, and ratio of stylet/body length in the five nymphal instars and adults of *Diaphorina citri* 1.

ACP instar	N	Stylet (µm)^2^	Body (µm)^3^	Ratio^4^
1st	31	266.0a	324.5a	0.828a
2nd	48	335.9b	529.4b	0.636b
3rd	38	397.8c	728.4c	0.547c
4th	25	503.9d	1135.1d	0.445d
5th	20	614.5e	1807.5e	0.342e
Adult	47	610.9e	2610.0f	0.244f

1 Means in the same column followed by the same letter are not significantly different (Tukey’s HSD test).

2 *F*_5, 165_ = 260, *P* = <0.0001.

3 *F*_5, 203_ = 3703, *P* = <0.0001.

4 *F*_5, 165_ = 161, *P* = <0.0001.

### Feeding Sites of ACP Nymphs on Young Citrus Leaves

Putative feeding sites of ACP nymphs on young healthy or LAS-infected ‘Valencia’ orange leaves were compared (Table 2). ANOVA on the proportion of nymphs that settled and presumably fed on each site (midrib or secondary veins, on upper or lower sides of the leaf) indicated that differences between healthy and LAS-infected leaves were not significant (*F* = 0.02, df = 1248, *P* = 0.88), whereas those among various sites were highly significant (*F* = 25.7, df = 3248, *P*<0.0001). A significantly higher proportion of nymphs was observed on the lower (abaxial) side of the leaf (64.5%) compared to those found on the upper (adaxial) side (35.5%) (χ^2^ = 54.7, *P* = <0.0001). Also, significantly more nymphs were observed on the midrib than on secondary/smaller veins (χ^2^ = 12.9, *P* = <0.0001), but that difference was much more pronounced on the upper side of the leaf than on the lower side (Table 2). On the midrib, the great majority of nymphs were observed on the sides (96.5%) rather than the top (center) of the midrib (3.5%) (Figs. 1B, C).

**Table 2:** Percentage of *Diaphorina citri* nymphs that settled (and putatively fed) on various sites of healthy/LAS-infected young ‘Valencia’ orange leaves^1^.

	Lower (abaxial) side		Upper (adaxial) side	
ACP instar	Midrib	Smaller veins	Midrib	Smaller veins
Healthy^2^	36.3a	32.0ab	23.4b	8.3c
Infected^3^	33.2a	25.3b	30.5ab	11.1c
Overall mean^4^	34.6a	28.3b	27.2b	9.8c

1 Results of two experiments. Means in the same row followed by the same letter are not significantly different, Ryan-Einot-Gabriel-Welsh multiple range test. Analyses conducted on arcsine-transformed percentages, raw data means presented.

2 *F*_3, 235_ = 18.6, *P* = <0.0001).

3 *F*_3, 251_ = 22.5, *P* = <0.0001).

4 *F*_3, 490_ = 36.9, *P* = <0.0001).

### Effects of Leaf age and LAS-infection on Feeding Sites and LAS Acquisition by ACP Adults

ANOVA on percentages of ACP adults that were observed presumably feeding on various sites of ‘Valencia’ orange leaves (Tables 3 &4) indicated the following putative feeding site preferences: 1) secondary veins were preferred over midribs, 2) the upper surface was preferred over the lower surface in healthy young leaves, 3) the lower surface was preferred over the upper surface in LAS-infected young leaves, and 4) adults consistently favored the lower surface of mature leaves especially in LAS-infected ones. No significant differences were found between the two experiments, replicate leaves, time or day of observation. In contrast to nymphs, most adults were observed presumably feeding on the top (center) rather than the sides of the midrib (Fig. 1D).

**Table 3:** Differences between putative feeding sites of *Diaphorina citri* adults on healthy and LAS-infected ‘Valencia’ orange leaves^1^.

	Mean percentage of adults feeding at each site			
Site on citrus leaf	Young healthy leaves^2^	Young infected leaves^3^	Mature healthy leaves^4^	Mature infected leaves^5^
Upper midrib	17.4c	11.8c	17.7c	3.5c
Upper sec. veins	42.8a	31.6b	33.4b	14.3bc
Lower midrib	11.0c	15.6b	7.9d	18.3b
Lower sec. veins	28.8b	41.1a	41.1a	64.0a

1 For each comparison, means in the same column followed by the same letter are not significantly different, least squares means.

2 *F*_3, 12_ = 77.9, *P* = <0.0001.

3 *F*_3, 12_ = 48.6, *P* = <0.0001.

4 *F*_3, 12_ = 92.9, *P* = <0.0001.

5 *F*_3, 12_ = 165.1, *P* = <0.0001.

**Table 4:** Effects of leaf age and LAS-infection on putative feeding sites of *Diaphorina citri* adults on ‘Valencia’ orange leaves^1^.

	Mean percentage of adults feeding at each site			
Leaf condition	Uppermidrib^2^	Uppersec. veins^3^	LowerMidrib^4^	Lowersec. veins^5^
Young healthy	16.9a	42.8a	11.0a	28.8b
Young infected	11.3a	31.5b	15.6a	41.1b
Mature healthy	17.2a	33.4b	7.9b	41.1b
Mature infected	2.7b	14.1c	18.6a	64.1a

1 For each comparison, means in the same column followed by the same letter are not significantly different, least squares means.

2 *F*_3, 12_ = 37.5, *P* = <0.0001.

3 *F*_3, 12_ = 48.3, *P* = <0.0001.

4 *F*_3, 12_ = 7.4, *P* = <0.0001.

5 *F*_3, 12_ = 33.1, *P* = <0.0001.

Based on qPCR tests following the second experiment, all the young and old infected leaves used tested positive for LAS, with an average Cq value of 20.66 and 20.59 for young and old leaves, respectively, with no significant differences in Cq value between them. However, the percentage of adults that acquired LAS from younger infected leaves with Cq thresholds of 36 or 40 (68.1 and 91.5%, respectively), was significantly higher than those acquiring it from mature infected leaves (2.1 and 29.8% respectively) (N = 47 for each, χ^2^ = 37.9, *P*<0.001). The mean Cq value for qPCR was also significantly lower (indicating higher LAS titer) in psyllids that fed on young infected leaves (mean Cq = 34.6), compared to those that fed on older infected leaves (mean Cq = 38.1) (*t* = 5.25; df = 55, *P*<0.001). None of the healthy control leaves or the psyllids that were caged on them tested positive for LAS in these qPCR tests.

### Structural Features and Parameters of Citrus Leaf Veins

**Figure 2:**
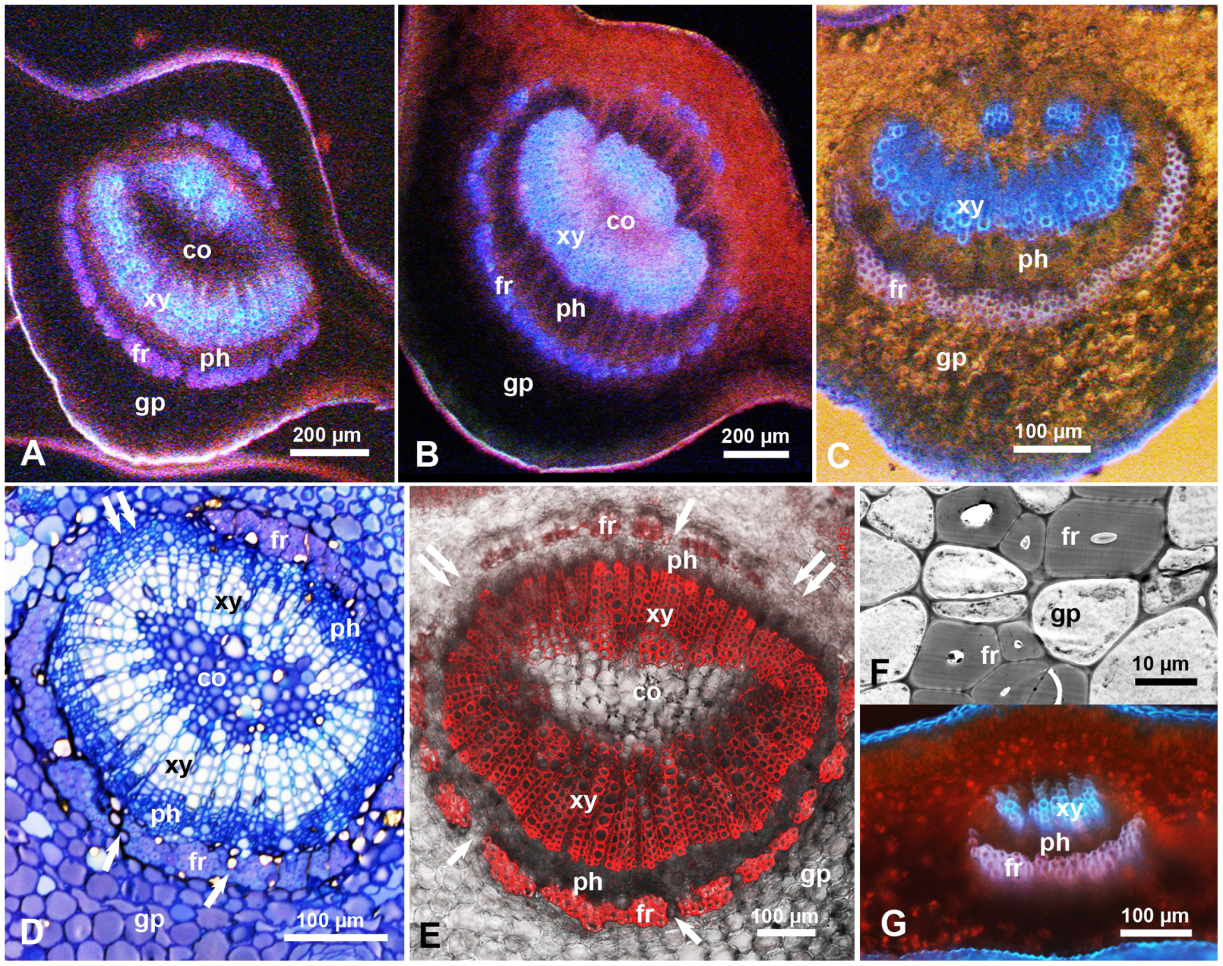
Cross sections in the midrib or secondary veins of ‘Valencia’ orange leaves. **A–C.** Sections in the midrib of mature healthy (A), mature Las-infected (B), and young Las-infected (C) leaves. **D & E.** Toluidine blue-stained semithin section (D) and confocal micrograph of a thicker (hand-cut) section (E) in the midrib of healthy leaves; single arrows indicate smaller gaps in the fibrous ring (fr); double arrows indicate wider gaps on the sides of the vascular bundle. **F**. Transmission electron micrograph showing thick-walled fibers of the fibrous ring (fr) with a small gap filled with ground parenchyma cells (gp). **G.** Section in a secondary vein of a healthy leaf. Leaf sections were examined with epifluorescence (A–C, G), light microscopy (D), confocal (E) or electron microscopy (F). Abbreviations: co, core cells; fr, fibrous ring; gp, ground parenchyma; ph, phloem; xy, xylem.

**Figure 3:**
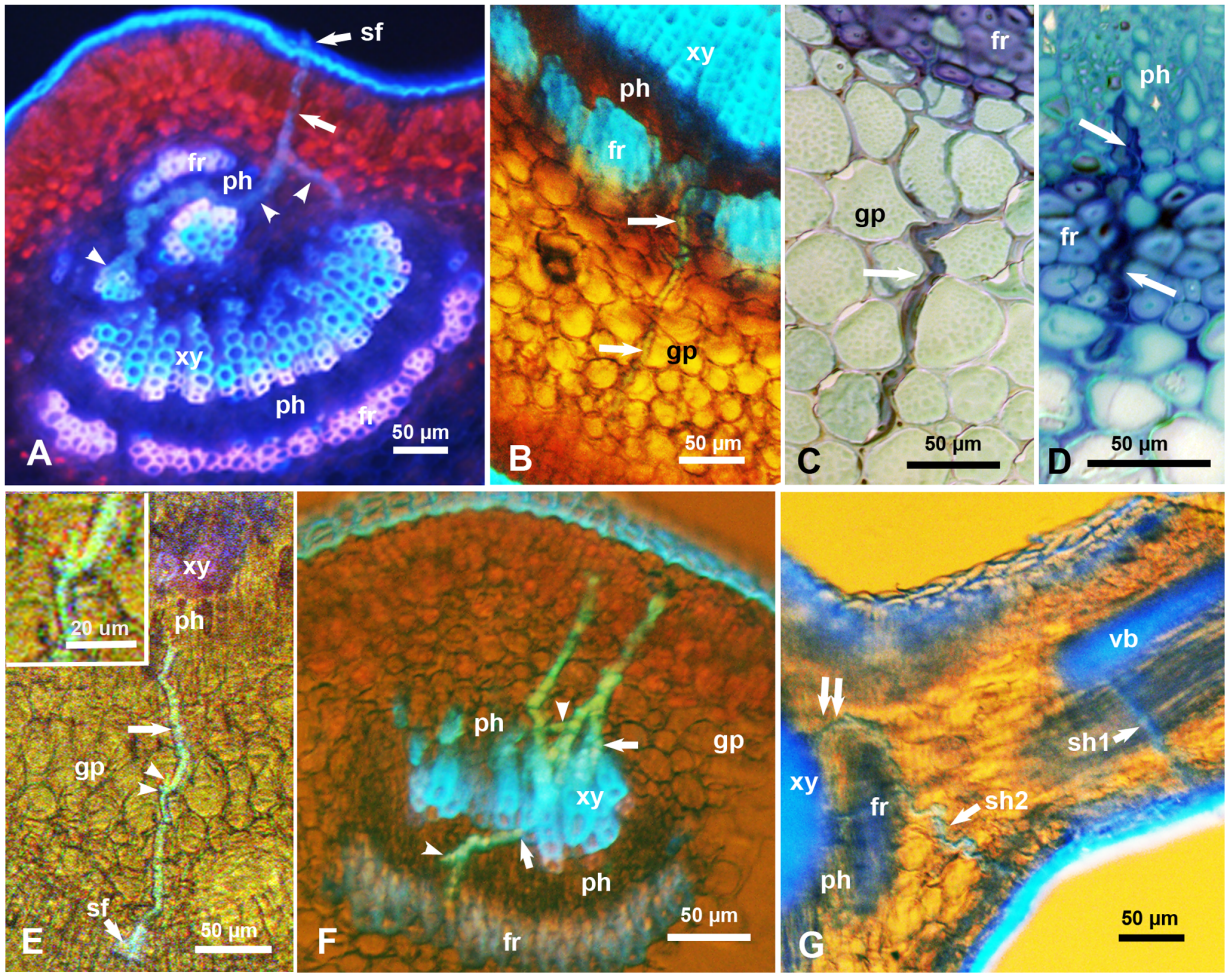
Salivary sheaths (unlabeled arrows) produced by psyllid adults (A–F) or nymphs (G), in the midrib or secondary veins of citrus leaves. **A & B.** Salivary sheaths entering from the upper (A) or lower (B) epidermis of the leaf; note that in both cases, these sheaths entered the phloem (ph) through gaps in the fibrous ring (fr), and (in A) branching of these sheaths in the phloem and xylem tissues (arrowheads). **C & D**. Toluidine blue-stained semithin sections in the midrib showing intercellular path of salivary sheaths in the ground parenchyma (in C) and in the fibrous ring (fr) before entering the phloem (in D). **E**. Salivary sheath entering the midrib from the lower side, going through the ground parenchyma (gp), with very short branches (arrowheads) in this tissue (inset) before entering the phloem. **F.** Three salivary sheaths entering a secondary vein from the upper or lower sides, some are branched in the phloem (arrowheads) and others apparently associated with xylem vessels (arrows). **G**. Two salivary sheaths, produced by nymphs, entering from the lower side of a young citrus leaf, one (sh1) entering the vascular bundle (vb) of a secondary vein, and the other (sh2) entering the midrib from the side/corner, then going around the fibrous ring (fr) to enter the phloem (ph) through a wide gap on the side of the vascular bundle (double arrows). Leaf sections were examined via epifluorescence (A), epifluorescence with transmitted light (B, E-G), or light microscopy (C, D). Abbreviations: co, core cells; fr, fibrous ring; gp, ground parenchyma; ph, phloem; sf, salivary flange; vb, vascular bundle; xy, xylem.

In cross sections in the midrib of mature or young citrus leaves (‘Valencia’ or ‘Ridge pineapple’ sweet orange), the upper (adaxial) and lower (abaxial) epidermis are followed by several layers of ground parenchyma around the vascular bundle (Figs. 2 & 3). Light, electron, epifluorescence and confocal laser scanning microscopy revealed that the phloem tissue is surrounded (from the outside) with an incomplete ring of thick-walled fibers (sclerenchyma) that will be termed here the ‘fibrous ring’ (Figs. 2A–G & 3A–D). In cross sections, this ring is composed of 1–5 layers of compact, donut-shaped fiber cells with very thick, apparently highly lignified, secondary walls (Figs. 2D–F). The fibrous ring is wider (with more layers) in the center, especially on the lower/abaxial side of the midrib, tapering towards the sides leaving a large gap (devoid of these fibers) on both sides of the vascular bundle (Figs. 2A–E). Several smaller gaps were also observed on both the lower/abaxial and upper/adaxial sides of the fibrous ring, and these gaps, in addition to the larger gaps on the sides, are occupied with much thinner-walled cells similar to those of the ground parenchyma (Figs. 2E, F). In the midrib of younger leaves, the fibrous ring on the upper side is less prominent, with fewer layers and wider gaps (Fig. 2C), compared to that in mature leaves (Figs. 2 A, B). In secondary veins, the fibrous ring on the upper side seems to be absent or rudimentary (Fig. 2G). Inside the fibrous ring of the midrib, the phloem tissue forms a second ring, followed by the xylem tissue which forms a third ring around a core of thin-walled cells apparently similar to those of the ground parenchyma (Figs. 2A–E). Here, the term ‘vascular bundle’ includes the fibrous ring, phloem, xylem, and the core inside the xylem tissue.

In cross sections of ‘Valencia’ orange leaves, the width of the phloem tissue and fibrous ring (at the center on the lower/abaxial side of the leaf), as well as the diameter of the vascular bundle, were greater in mature than in younger leaves, in the midrib compared to the secondary veins, and in LAS-infected compared to healthy leaves (Table 5, Figs. 2A–C). Also, the ratio of the width of both the phloem and fibrous ring compared to the diameter of the vascular bundle were greater in infected than in healthy leaves and in the midrib than in secondary veins. ANOVA indicated that most of these differences were significant (Table 5). Similarly, the distance (from the leaf surface) to the phloem was significantly greater in LAS-infected than in healthy leaves (209.2 and 157.2 µm, respectively), in mature than in younger leaves (238.0 and 136.8 µm, respectively), in the midrib compared to the secondary veins (243.2 and 95.1 µm, respectively), on the lower than on the upper side of the leaf (223.3 and 153.2 um, respectively), and from the center/top compared to the side of the veins (209.2 and 167.2 um, respectively). Results of the pair-wise comparisons among all of these sites are shown in Table 6.

**Table 5:** Width of the phloem tissue and fibrous ring, and their ratio to vascular bundle diameter in the midrib and secondary veins of healthy/LAS-infected, and young or old, ‘Valencia’ orange leaves (all measurements in µm)^1^.

Comparisonbetween	Phloem width	Fibrous ringwidth	Vascularbundle diam.	Phloem/vascular bundle	Fib. ring/vascular bundle
Mature leaf	109.2a	40.4a	589.6a	0.20a	0.15a
Young leaf	43.1b	30.4b	226.4b	0.20a	0.09b
Midrib	94.8a	37.3a	574.9a	0.25a	0.19a
Second veins	45.9b	32.4b	178.0b	0.17b	0.08b
Las-infected	99.9a	37.1a	464.8a	0.23a	0.14a
Healthy	42.3b	32.9a	345.4b	0.16b	0.11b

1 For each comparison, means in the same column followed by the same letter are not significantly different (*t* tests, *P*<0.05).

**Table 6:** Mean distance to the phloem from the center (top) or sides of the midrib and in secondary veins of healthy/LAS-infected, young or old ‘Valencia’ orange leaves.

			Distance to the phloem (µm)^1^			
	Leaf category		Upper (adaxial) side		Lower (abaxial) side	
Vein category	Age	Las/HLB	Center	Side	Center	Side
Midrib	Young	Healthy	143.5a	118.2a	237.0a	192.4a
		Infected	129.6a	115.0a	259.1a	200.1a
Midrib	Old	Healthy	268.0a	175.5a	312.8a	224.4a
		Infected	315.5b	244.4b	448.5b	301.8b
Secondary veins	Young	Healthy	59.8a	65.3a	77.7a	82.2a
		Diseased	70.4b	76.8a	108.7b	116.4b
Secondary veins	Old	Healthy	89.6a	96.1a	121.7a	134.0a
		Infected	72.7b	80.4b	131.7a	154.1a

1 For each vein and leaf category combination, means in the same column followed by the same letter are not significantly different (*t* tests, *P*<0.05).

### Salivary Sheaths of ACP Nymphs and Adults

Salivary sheaths, confirming feeding by both nymphs and adults of ACP, were examined using both light and epifluorescence microscopy (Figs. 3A-G). These sheaths were found in the midrib or secondary veins of sweet orange leaves (‘Valencia’ or ‘Ridge pineapple’) at or close to the observed putative feeding sites mentioned above. The salivary sheaths were ‘gnarled’ consisting apparently of ‘bursts’ of secretions along their route; most were found to branch, especially inside the phloem tissue (Figs. 3A, F). These sheaths normally were found to take a slightly tenuous course through the ground parenchyma layers, then through the fibrous ring, before branching and terminating mainly in the phloem tissue (Figs. 3A-G) although some sheaths appeared to terminate in xylem vessels (Figs. 3A, F). Out of 41 salivary sheath termini produced by ACP adults that reached a vascular bundle, 33 (80. 5%) were associated with the phloem and 8 (19.5%) were associated with xylem vessels. Also, out of 22 salivary sheath termini produced by ACP nymphs (Fig. 3G) that reached a vascular bundle, 20 (91%) were associated with the phloem while only 2 (9%) were associated with xylem vessels. The difference in this regard between nymphs and adults sheath termini was not significant (χ^2^ = 1.164, *P* = 0.281).

The salivary flange was sometimes observed on the outer portion of the salivary sheath where it entered the upper or lower leaf surface (Figs. 3A, E). Most of the salivary sheaths of nymphs started on the sides rather than the center of the midrib, especially on the lower side of the leaf (Fig. 3G). However, sheaths of adults started anywhere along the veins, including the center or sides of the midrib or secondary veins. In several cases, the salivary sheaths of nymphs or adults were observed to enter the phloem from the cortical parenchyma through one of the wider or smaller gaps in the fibrous ring (Figs. 3A, B). In other cases, the salivary sheaths seemed to bend around the fibrous layer then enter the phloem through the larger gaps on either side of the vascular bundle (Fig. 3G).

In semithin (1–2 µm) sections of the midrib, the path of the salivary sheaths in the ground parenchyma and fibrous ring appeared to be intercellular (Figs. 3C, D). However, epifluorescence of thicker (50–70 µm) sections showed very short branches (5–10 µm long) of some salivary sheaths in the ground parenchyma (Fig, 3E and its inset), suggesting that psyllids may be sampling these cells along the way before they reach the phloem or xylem. Branches of the salivary sheaths that terminated in the phloem tissue were much longer, reaching 232 µm (Figs. 3A, F, G), which is more than one third of the adult stylet length.

### Ultrastructure and Width of ACP Maxillary Food and Salivary Canals and HLB-Associated Bacteria

**Figure 4:**
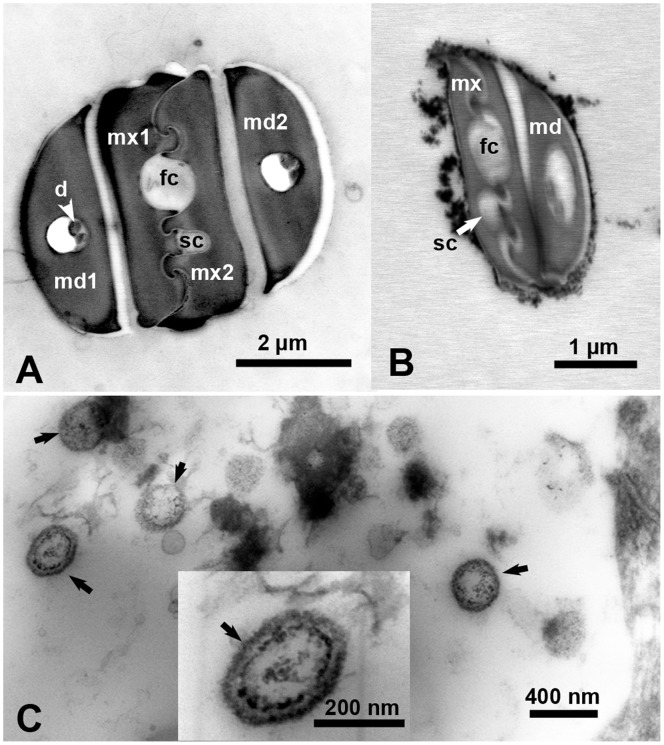
Electron micrographs of ultrathin cross sections in the stylets of a psyllid adult (A), stylets of 1^st^ instar nymph (B), and in the phloem of a LAS-infected citrus leaf (C). Unlabeled arrows (in C) indicate bacterial structures found in the phloem of infected leaves. Abbreviations: d, dendrites; fc, food canal; md1&2, mandibular stylets 1& 2; mx1&2, maxillary stylets 1 & 2; sc, salivary canal.

Ultrathin sections of the stylets in ACP adults and 1^st^ instar nymphs were examined by TEM, and the width of the food and salivary canals, formed by the two interlocking maxillary stylets (Figs. 4A, B) were measured. Width of the food canal averaged 817.5±62.5 µm in the adults and 460.0±37.6 µm in 1^st^ instar nymphs (*t* = 19.4, df = 57, *P* = 0.000). The width of the salivary canal averaged 379.5±34.7 µm in the adults and 212.0±17.9 µm in 1^st^ instar nymphs (*t* = 18.8, df = 57, *P* = 0.000). In thin sections of LAS-infected leaves (Fig. 4C), the width of quasi-spherical, double-membrane bound bacteria-like organisms found in the phloem ranged between 233–314 nm, with a mean width of 279±45.7 nm. These organisms were not found in thin sections in the phloem of healthy citrus leaves.

## Discussion

### Stylet Length in Relation to Feeding Sites and Vein Structure

It has been known for several years that older nymphs of ACP are more efficient than adults in transmission of the phloem-limited LAS bacteria strongly associated with HLB, currently the most devastating citrus disease worldwide [4], [5], [6]. The fact that younger ACP nymphs are known to feed mainly and to survive best on young citrus tissue compared to mature leaves [19], [20] in addition to previous reports that 1^st^ and 2^nd^ instar nymphs cannot acquire and/or inoculate LAS/HLB [4], [5], raise the question whether the stylets of younger ACP nymphs are long enough to reach the phloem of citrus leaves. Most of the previous studies on feeding behavior and stylet morphometrics of ACP and other psyllids were done on adults rather than nymphs [17], [18], [25], probably because of the smaller size and fragile nature of psyllid nymphs especially during early instars. The finding that almost all exuvia in psyllids and other hemipterans have fully extended stylets [21] has made it easier to measure the stylets of various nymphal instars. In the present work, when comparing the stylet length of 1^st^ to 5^th^ instar nymphs of ACP with the distance to the phloem in citrus leaves, we can conclude that even the youngest nymphs of ACP should be able to successfully feed on the phloem of both young and old leaves. However, the fact that salivary sheaths of both nymphs and adults were found to frequently branch, sometimes for a long distance (up to one third of the stylet length), in the phloem (Figs. 3A, F, G) [25] suggests that the stylets not only need to reach the phloem but must also be able to ‘wander’ inside the phloem tissue, probably contacting and sampling different sieve elements looking for suitable or sufficient nutrients.

Technically, only EPG can definitively identify ‘feeding’ in real-time, i.e. when a hemipteran’s stylets are inserted in a plant [14], [16], [18]. Yet, it is reasonable to assume that plant sites where ACP adults or nymphs settle and attain their typical feeding postures also represent putative feeding sites, especially that we verified this later by microscopy of extended stylets and histology of citrus leaves as mentioned in the Methods section. Our results with citrus leaf vein structure show that the distance to the phloem was significantly shorter in younger than in mature leaves, shorter from the sides of the midrib than from its top/center, shorter from the upper than the lower surface of the leaf, as well as shorter in smaller veins than in the midrib. These results may explain the fact that ACP nymphs settle and feed almost exclusively on young citrus leaves, and also why they do so mainly on the sides rather than the center of the midrib. However, our results do not explain the preference of nymphs to settle and feed on the lower side of citrus leaves, or on the midrib especially of the upper surface of the leaf. It is possible that both the midrib and the lower leaf surface may afford more protection for these fragile and docile nymphs from their natural enemies and/or climatic conditions (e.g. rain, sunshine etc.). Additionally, or alternatively, nymphs may prefer the lower leaf surface because their honeydew excretions will normally drop from the leaf rather onto it, which can be seen when comparing Figs. 1B and 1C.

Contrary to the behavior of nymphs, ACP adults preferentially settled and fed on secondary veins, rather than the midrib, on the upper or lower sides of citrus leaves. Most of the salivary sheath termini by nymphs and adults were associated with the phloem, whereas only 10–20% of the sheath termini were associated with the xylem. Based on EPG results, Bonani et al. [16] also suggested that a small proportion of ACP adults may be probing the xylem, and Cen et al. [18] indicated that in diseased citrus plants more ACP adults may probe the xylem than in healthy plants. Some of our micrographs (Fig. 3C) show clearly an intercellular path of ACP salivary sheaths within the ground parenchyma, which was also suggested by Bonani et al. [16] who observed ‘intact’ epidermal and parenchyma cells surrounding the salivary sheaths of adult ACP. However, our observation of very short branches of salivary sheaths in the ground parenchyma (Fig. 3E) suggests that psyllids may also be sampling these parenchyma cells along the way before reaching the phloem.

### Role of the ‘Fibrous Ring’ around the Phloem

In addition to the stylet length or distance to the phloem, another important factor in determining the feeding behavior of ACP nymphs and adults seems to be the ‘fibrous ring’, composed of a few layers of compact thick-walled fibers surrounding the phloem tissue. This structure has been referred to in earlier works as sclerenchyma fibers [26], sclerenchymatic sheath [27], phloem fibers [28], undifferentiated pericyclic fibers [16] and fiber strand [29]. Richter et al. [30], studying *Phormium tenax* leaf tissues, observed the highest lignification in the cell corners and cell walls of the sclerenchyma fibers surrounding the vascular tissue, and suggested that this lignification can act as a protective barrier for the vascular tissue. Our results show that the fibrous ring in citrus leaves is more prominent (with more layers and fewer or narrower gaps) in mature than in young leaves, in the center than on the sides of the midrib, and on the lower than the upper side of the leaf, and is rudimentary or absent in the upper side of secondary veins. These findings can explain the preference of nymphs to settle and feed on younger over mature leaves and from the sides compared to the top/center of the midrib. They can also explain the adults’ preference to settle and feed on the secondary veins rather than the midrib on both the upper and lower leaf surfaces.

In our work, we observed that several salivary sheaths of both adults and nymphs either had penetrated through one of the small gaps along the fibrous ring or around this ring to enter the phloem from much wider gaps on either side of the vascular bundle. As we indicated, these gaps are occupied by thinner walled cells similar to those of the ground parenchyma. This tissue is probably easier to traverse intercellularly than the compact thick-walled cells of the fibrous ring. It is possible that, in other instances, ACP stylets can penetrate between cells of the fibrous ring (Fig. 3D), although they probably prefer to go through those gaps if they found one nearby. It is also possible that ACP adults may be more able than nymphs to overcome the thicker fibrous ring, either by going through or around it to reach the phloem. Bonani et al. [31] reported that, within 5 h, 50% of adult ACP adults started phloem ingestion on young citrus leaves whereas only 15% ingested from phloem on mature leaves. The authors proposed that these differences may be due to a thicker layer of fiber cells in mature leaves, reducing phloem feeding. Furthermore, Rangasamy et al. [32] studied the distribution of salivary sheaths of the chinch bug (*Blissus insularis* Barber (Hemiptera: Blissidae) and axillary shoot lignification in resistant and susceptible cultivars of St. Augustinegrass *Stenotaphrum secundatum* (Walter). Salivary sheaths were more abundant on the outermost leaf sheath of axillary shoots of resistant cultivars compared with susceptible ones. These results, combined with electron microscopy, suggested that the thick-walled sclerenchyma cells around the vascular bundle play a role in southern chinch bug resistance in St. Augustinegrass, possibly by reducing stylet penetration to the vascular tissue [32]. The possible role of the fibrous ring in resistance of some citrus cultivars/lines against ACP should be investigated and may be useful for designing more resistant lines against ACP and/or HLB.

### ACP Feeding Sites and Vein Structure in Healthy vs. LAS-infected Citrus Leaves

We found no significant differences in feeding sites of ACP nymphs between healthy and LAS-infected young citrus leaves. However, ACP adults appeared to differ in this regard according to the leaf age, since they preferred to settle and feed on the lower secondary veins in LAS-infected (young or old) citrus leaves and on the upper secondary veins only in young healthy leaves (Table 3). Using various microscopy techniques, we showed that in cross sections of healthy and diseased (LAS-infected) citrus leaves, the width of the phloem and fibrous ring, and their ratio to the vascular bundle diameter were greater in diseased than in healthy leaves (Table 5, Figs. 2A–C). Using transmission electron microscopy, Folimonova & Achor [28] also observed “the enlargement of the phloem layer” in LAS-infected citrus leaves, and suggested that the increased number and size of phloem parenchyma may account for this phloem enlargement. Greater width of the phloem tissue in HLB-diseased than in healthy citrus leaves can also be seen in Figs. 2A & 2B by Kim et al. [33]. These authors indicated that HLB bacterial infection caused phloem disruption, sucrose accumulation, and plugged sieve pores. The HLB-associated phloem blockage is thought to have resulted from the deposition of large amount of callose around the sieve pores rather than the HLB bacterial aggregates, because LAS does not form aggregates in citrus [29], [33]. Folimonova & Achor [28] suggested that one of the first degenerative changes induced upon invasion of the pathogen appears to be swelling of middle lamella between cell walls surrounding sieve elements. Thus, the thicker/wider phloem area reported here may be due to this ‘swelling’ of the middle lamella of sieve elements and/or, possibly, to the LAS-infected plants producing more phloem cells to compensate for the clogged ones, as suggested by Cen et al. [18].

EPG studies with ACP adults indicated that, in LAS-infected citrus plants, the times to first and sustained salivation in the phloem were longer than those in healthy plants. Also, as symptom expression increased, the percentage of time spent by psyllids putatively salivating during the phloem phase increased, whereas the percentage of time spent in all phloem activities (salivation and ingestion) was reduced [18]. Thus, Cen et al. [18] suggested that, in LAS-infected plants, these effects may be due to the phloem being a less suitable food source or the psyllid being less able to overcome phloem wound responses prior to ingestion. It is has not been possible to ascertain, via EPG alone, in which cell types these activities occur. Thus, it is possible that the fibrous ring around the phloem, which is thicker in diseased than healthy leaves as we showed here, may be at least partly responsible for the increased time before the first and sustained salivation in the phloem by ACP adults.

In our study a much higher percentage of ACP adults were able to acquire LAS from younger infected citrus leaves compared to mature leaves, even though both types of leaves had similar titers of LAS bacterium. This may be due to the longer period of sustained feeding in the phloem by ACP on younger leaves [31]. Alternatively, or additionally, it is possible that younger leaves may harbor more live than dead LAS bacteria which cannot be distinguished in most PCR tests currently used. Using TEM, Folimonova et al [28] did not observe the HLB-associated bacteria in highly symptomatic leaf samples, suggesting a possibility that, at more advanced stages of the disease, a major proportion of LAS is present in a nonviable state.

### The Maxillary Food and Salivary Canals as Possible Barriers to HLB Bacterium

The piercing sucking mouthparts of ACP and other hemipterans are composed mainly of the labrum, labium and stylet bundle [34]. The latter is composed of four stylets, two mandibular (on the outside) and two maxillary ones (on the inside) that interlock to form two canals, with the (larger) food canal for sucking plant juice, and the (smaller) salivary canal for injection of salivary secretions (Figs. 4 A, B) [34]. The phloem-limited HLB/LAS bacterium is transmitted by ACP in a persistent manner, which means that it is acquired by ACP while feeding on LAS-infected plants though the maxillary food canal, and, following a short latent period in the vector, is inoculated into other citrus plants with salivary secretions that pass through the maxillary salivary canal. Garzo et al. [17], who studied the mouthparts of ACP adults only, reported that the width of the food and salivary canals in adults is 0.9 and 0.4 µm, respectively. In our study, width of the food and salivary canals for adults was 818 nm (0.82 µm) and 380 nm (0.38 µm) respectively which is very close to that reported by Garzo et al. [17]. Additionally, in 1^st^ instar nymphs, we found that the width of the food and salivary canals were 460 and 212 nm, respectively, i.e. nearly half the width of those of the adults. Diameter of the bacteria that we found in the phloem of LAS-infected leaves averaged 279 nm, which is within the range of reported diameters of HLB-associated bacteria 200–300 nm [35], 330–660 nm [36], and 130–430 nm [28]. It is clear that the width of the food canal in both ACP adults and 1^st^ instar nymphs is wide enough to accommodate HLB-associated bacteria during ingestion (and Las acquisition). Although the width of the salivary canal in adults is large enough to accommodate such bacteria through during salivation (and Las inoculation), that of 1^st^ instar nymphs is apparently not wide enough for most of these bacteria. This indicates that the width of the salivary canal in 1^st^ instar nymphs can be a barrier to inoculation of LAS/HLB, which may explain previous reports that younger (1^st^ and 2^nd^ instar) nymphs cannot acquire and/or inoculate LAS/HLB from and to citrus plants [4], [5], [6]. Since HLB associated bacteria have double membranes [1], they are expected to be less flexible than single-membrane bound phytoplasma and spiroplasma, which take much more bizarre shapes in plants, insects and in culture [23], [37].

### Conclusions

1. Morphometrics of the stylets of ACP nymphs indicated that the youngest instars can reach and feed in the phloem on both sides of young citrus leaves, especially on the secondary veins or on the sides of the midrib.
2. The majority (80–90%) of the salivary sheaths termini produced by ACP adults or nymphs that reached a vascular bundle were associated with the phloem whereas only 10–20% were associated with xylem vessels.
3. Although the width of the maxillary food canal in first instar nymphs is wide enough for LAS bacteria to go through during food ingestion (and LAS acquisition), the width of the salivary canal in these nymphs may not be wide enough to accommodate LAS bacteria during salivation (and LAS inoculation) into host plants. This may explain the inability of early instar nymphs to transmit LAS/HLB in earlier reports.
4. The thick-walled ‘fibrous ring’ (sclerenchyma fibers) around the phloem in citrus leaves, which is more prominent in older than in younger leaves and in the center than on the sides of the midrib, may be a barrier to ACP stylet penetration to the phloem. The possible role of this fibrous ring in resistance of some citrus cultivars/lines against ACP or other psyllids should be investigated and may be useful for designing more resistant lines against ACP and/or HLB.
